# Comprehensive Analysis of Drug-Induced Parkinson-like Events

**DOI:** 10.3390/ph17081099

**Published:** 2024-08-22

**Authors:** Mami Kikegawa, Hideko Sone, Yoshihiro Uesawa

**Affiliations:** 1 Department of Medical Molecular Informatics, Meiji Pharmaceutical University, 2-522-1 Noshio, Kiyose 204-8588, Japan; m.takigawa@yok.hamayaku.ac.jp; 2 Department of Kampo Medicine, Yokohama University of Pharmacy, Yokohama 245-0066, Japan; 3 Environmental Health and Prevention Research Unit, Department of Health Science, Graduate School of Pharmaceutical Sciences, Yokohama University of Pharmacy, Yokohama 245-0066, Japan; hideko.sone@yok.hamayaku.ac.jp

**Keywords:** Parkinson-like events, Japanese Adverse Drug Event Report database, spontaneous reporting system, volcano plot, hierarchical clustering, principal component analysis

## Abstract

Specific drugs are well known to have the capacity to induce Parkinson-like symptoms. Parkinson-like events are side effects that may persist for an extended period even after drug administration is discontinued. Although these events can be triggered by various drugs, the mechanisms underlying their diverse symptoms remain largely unclear. To investigate this, we used the Japanese Adverse Drug Event Reporting Database, which is maintained by the Pharmaceuticals and Medical Devices Agency, to analyze the risk factors associated with Parkinson-like events along with the associated drug trends and characteristics. Our findings indicate that similar to Parkinson’s disease, age-related differences affect the onset of these reported events, with older individuals being more susceptible. Hierarchical clustering and principal component analysis revealed that the mechanisms triggering these Parkinson-like events are consistent across reports, suggesting a common underlying cause. However, even with a consistent mechanism, the side effects can vary depending on the site of action. These insights underline the importance of the swift identification of the drugs suspected of causing these events and the implementation of measures to reduce their side effects.

## 1. Introduction

Parkinson’s disease is a neurodegenerative disease that develops because of relatively selective damage to dopamine neurons in the substantia nigra and is characterized by motor symptoms centered on bradykinesia (slowness of the initiation of voluntary movement with reduced speed and range of repetitive actions), tremors, and muscle rigidity. Furthermore, its prevalence in Japan is estimated to be 0.1–0.18%, and aging contributes to its onset [1]. In contrast, antipsychotics have dopamine receptor-blocking effects and cause drug-induced Parkinsonism [2]. Parkinsonism is defined as a pathological condition characterized by various symptoms similar to those observed in Parkinson’s disease. Among these disorders, types in which Parkinson’s disease symptoms manifest as the side effects of medications were classified as drug-induced Parkinsonism. Moreover, conditions that exhibited Parkinson-like symptoms but were not categorized as Parkinson’s disease were referred to as Parkinson’s syndrome. Drug-induced movement disorders could be associated with therapies that involve levodopa or dopamine agonists or to drugs possessing central dopamine receptor antagonist properties, anticholinergics, certain anticonvulsants, and amphetamines. These disorders typically arise from lesions or dysfunction within the extrapyramidal motor system (a part of the motor network that controls involuntary movements), leading to impairments in motor function. The manifestations of these disorders can closely mimic those of idiopathic Parkinson’s disease, featuring the classic triad of resting tremors, rigidity, and bradykinesia. Additional Parkinson-like symptoms may include bradykinesia (reduced speed of movement), hypersalivation, drooling, shuffling gait (dragging steps), micrographia (abnormally small and cramped handwriting), dysphonia (abnormal voice quality or volume), and diminished postural reflexes [3]. Approximately 30% of patients with schizophrenia have been reported to have a poor response to antipsychotic drugs. Seeking to improve positive symptoms, clinicians may resort to prescribing higher doses or combinations of multiple drugs, which can increase the likelihood of side effects and treatment resistance. Maintaining the optimum dosage of antipsychotic drugs and balancing the therapeutic effects and side effects are important. In drug-induced Parkinsonism (DIP), Parkinson’s disease symptoms appear when approximately 80% of dopamine receptors 2 (D2-R) are blocked [4]. The Manual for Handling Serious Side Effects and Diseases of the Ministry of Health, Labour, and Welfare lists drugs with many side effects. However, clozapine and quetiapine have a blocking effect of approximately 60%; therefore, side effects are unlikely to occur [5]. When DIP occurs, the only option is to reduce the dose of the causative drug and, in severe cases, temporarily discontinue it. However, many patients with DIP may continue to have Parkinsonian symptoms long after discontinuation of the causative drug, suggesting potential neurodegeneration [6]. Parkinson-like events have a wide-ranging impact on the patients’ quality of life. Although these symptoms themselves are rarely a direct cause of death, they increase the risk of various complications, which can be life-threatening. Therefore, DIP is considered a significant side effect and is extremely important for comprehensively analyzing the relationship between Parkinson-like side effects and the drugs that induce them. The Japanese Adverse Drug Event Reporting (JADER) database, which is published by the Pharmaceuticals and Medical Devices Agency (PMDA), reports various side effects of drugs, including Parkinsonism, bradykinesia, tremors, and muscle rigidity. It has been reported as a “Parkinson-like event”. Although this disease was induced by various drugs, the mechanisms underlying its various symptoms have not been fully elucidated. To date, investigations on Parkinson-like events have been limited to simple counts of side effects or case reports involving specific drugs. However, the mechanisms underlying these symptoms have not been fully elucidated. Therefore, in this study, we comprehensively analyzed the relationship between Parkinson-like events and the drugs that induce them using the JADER database.

## 2. Results

### 2.1. Preparing Data Tables for Analysis

**Figure 1:**
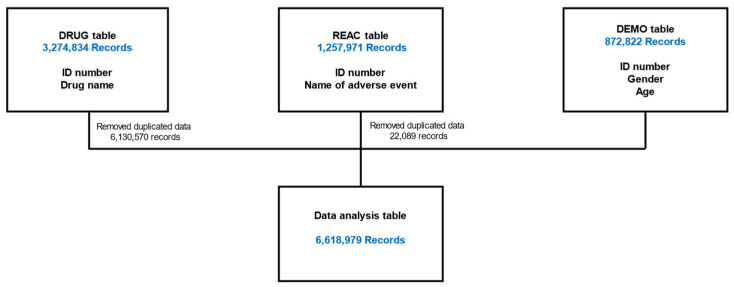
Flowchart for the construction of the data analysis tables. DRUG, drug information; REAC, adverse reaction information; DEMO, patient demographic information. Duplicate data in the DRUG tables were deleted. Three tables were combined using identification numbers.

Figure 1 shows a flowchart for creating a data table for analysis. The data were extracted from the drug information (DRUG) table (3,274,834 records), adverse reaction information (REAC) table (1,257,971 records), and patient demographic information (DEMO) table (872,822 records) from the JADER database. We eliminated two duplicate tables and combined them, resulting in 6,618,979 records in a final data table.

### 2.2. Relevance of Parkinson-like Events to Patient Characteristics

Table 1 shows the characteristics of the patients with Parkinson-like events. In the Parkinson-like events patient group, statistical analysis revealed a significant association with sex (*p* < 0.0001) and age (*p* < 0.0001). Females were 1.287 times more likely to develop Parkinson-like events than males. Furthermore, individuals aged ≥40 and ≥70 years were 1.562 and 1.524 times more likely to develop Parkinson-like events, respectively.

### 2.3. Relationship between Parkinson-like Events and Drugs

**Figure 2:**
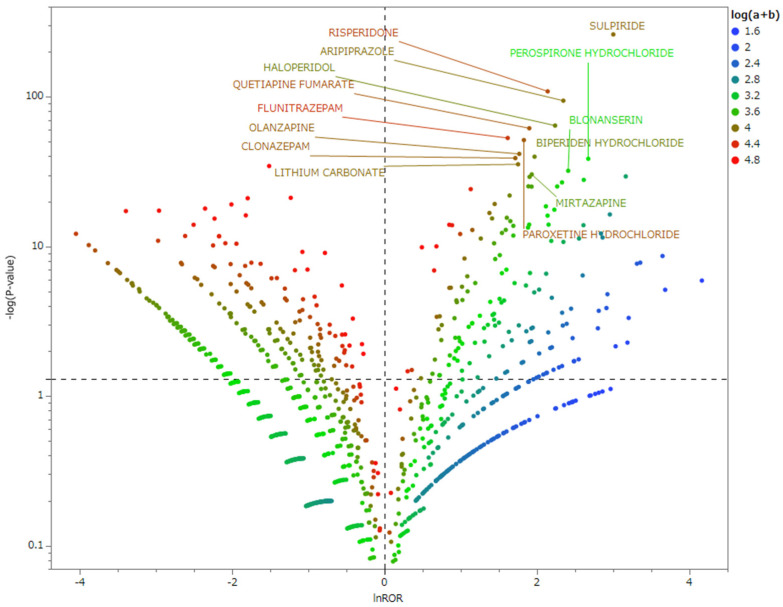
Drugs associated with Parkinson-like events. The X-axis shows the natural logarithm of the reported odds ratio (ln ROR). The Y-axis shows the reciprocal of the common logarithm for the *p*-value of Fisher’s exact test [−log(*p*-value)]. RORs were calculated using a crosstabulation table. The dotted line on the Y-axis represents a *p*-value of 0.05. The color of the plot represents the number of reported adverse events. Higher common logarithms of the total reported counts are shown in red and lower ones in blue. The color of the plot represents the number of reported adverse events. The common logarithm of the total number of reported times is shown in red to blue.

Figure 2 shows the association of Parkinson-like events with drug use. The drugs plotted in the upper right tend to induce Parkinson-like events. The drugs labeled in Table 2 are displayed in the scatter plot.

Table 2 shows the reporting odds ratios for the association between high-risk drugs and Parkinson-like events. High-risk drugs with over 10,000 reports of DIP include sulpiride, aripiprazole, risperidone, and quetiapine fumarate. These drugs have RORs and 95% confidence intervals (CI) as follows: sulpiride, 20.10 (CI: 17.87–22.60); aripiprazole, 10.44 (CI: 8.88–12.28); risperidone, 8.50 (CI: 7.38–9.79); and quetiapine fumarate, 6.68 (CI: 5.63–7.93). The other drugs listed in the reports of serious adverse reactions with significant odds ratios for DIP include blonanserin, paroxetine hydrochloride, and several benzodiazepines—specifically mirtazapine, clonazepam, and flunitrazepam—with respective values of 11.13 (95% CI: 8.37–14.78), 6.22 (95% CI: 5.19–7.47), 6.90 (95% CI: 5.39–8.84), 5.56 (95% CI: 4.56–6.79), and 5.03 (95% CI: 4.28–5.92), respectively. All 14 high-risk drugs that met the statistical significance conditions of ln (ROR) > 1.5 and −log(*p*-value) > 30 had *p*-values of <0.0001, indicating a very strong association with DIP.

### 2.4. Hierarchical Clustering of Parkinson-like Events

**Figure 3:**
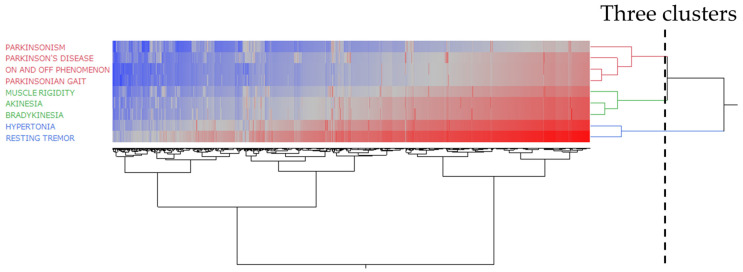
Hierarchical clustering of Parkinson-like events. The classification of drugs associated with Parkinson-like events using hierarchical clustering. The dendrogram shows the relationship between 2064 drugs and 9 side effects related to Parkinson-like events. Based on a hierarchical cluster analysis, the side effects were classified into three groups and colored red, blue, and green. The color map shows the correlation between the variables. The larger the lnROR value, the redder it appears, and the smaller the value, the bluer it appears.

Figure 3 shows a dendrogram generated by hierarchical clustering resulting in three clusters. Based on the hierarchical cluster analysis of the side effects, the results were categorized into three groups: (1) Parkinsonism, Parkinson’s disease, the on–off phenomenon, and Parkinsonian gait; (2) muscle rigidity, akinesia, and bradykinesia; and (3) hypertonia and resting tremor.

### 2.5. Star Dendrogram Associated with Parkinson-like Events

**Figure 4:**
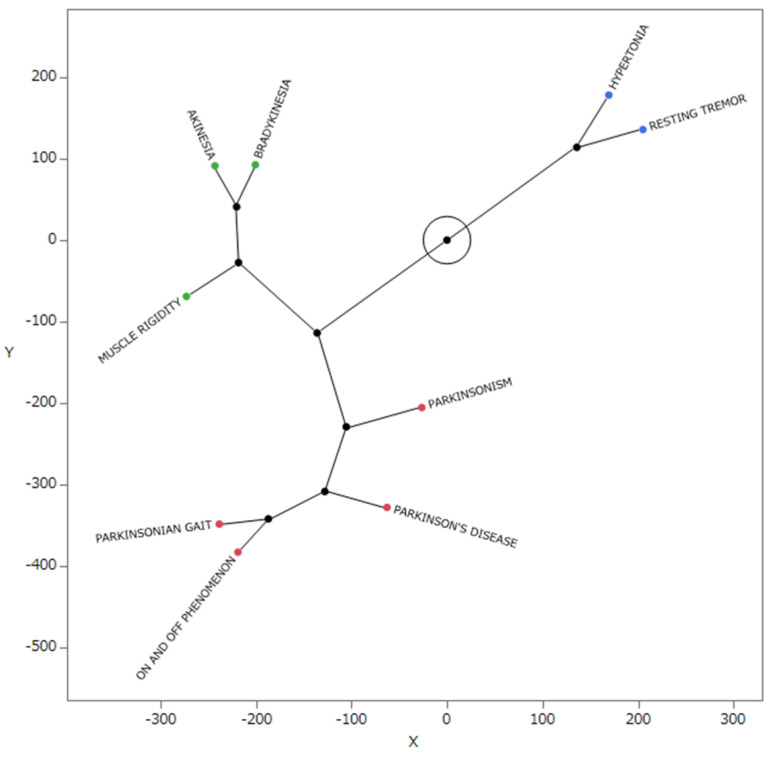
Star dendrogram associated with Parkinson-like events. A classification of drugs associated with Parkinson-like events was generated in the hierarchical clusters. The colored points represent side effects, which were classified into three groups based on a hierarchical cluster analysis. Furthermore, the star dendrogram shows the relationship between the nine types of side effects associated with Parkinson-like events. We represent side effects as the endpoints and each cluster connection as a new point. The lines represent membership within a cluster. The length of the line between cluster bonds represents the approximate distance between the bonded clusters.

Figure 4 presents a star dendrogram generated by the hierarchical clustering that resulted in three clusters. Muscle hypertonia and resting tremor were isolated from the other side effects, showing a pattern distinct from that observed in the other categories.

### 2.6. Principal Component Analysis (PCA) Shows Association between Parkinson-like Events and Drugs

**Figure 5:**
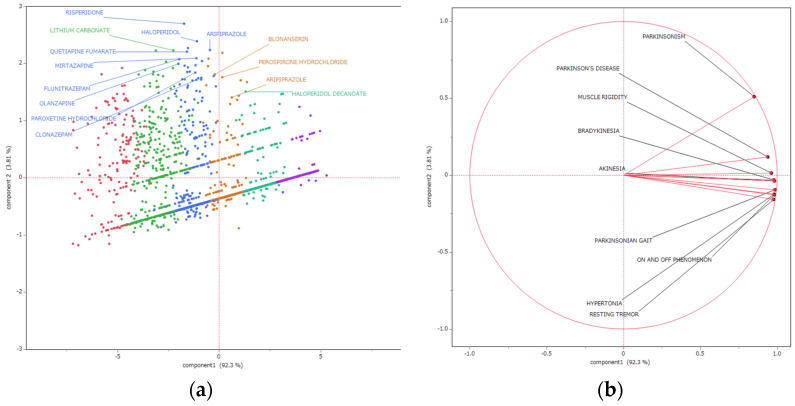
Association between Parkinson-like events and pharmaceuticals using PCA. The principal component score plot (**a**) shows the relationship between the pharmaceuticals and each principal component. Each plot represents drugs. The colors of each plot were determined by dividing the 2,064 drugs into six categories using hierarchical clustering. The principal component loading plot (**b**) shows the relationship between Parkinson-like events and each principal component. Each loading vector represents an adverse event.

Figure 5 shows the results of the PCA. The contribution of the principal components was 92.3% for principal component 1 and 3.81% for principal component 2. We created a scattergram using the principal components 1 and 2 (Table S1). We then visualized the relationship between Parkinson-like events and each principal component representing each adverse event as a loading vector. In the loading plot, each adverse event was concentrated toward the right side and distributed very closely. Only Parkinsonism was slightly separated and positioned above the other groups. Meanwhile, the score plot shows 14 drugs as high-risk drugs, characterized by having an ln (ROR) > 1.5 and a −log(*p*-value) > 30.

## 3. Discussion

### 3.1. Parkinson-like Events and Patient Characteristics

In this study, we observed significant differences in the occurrence of reported Parkinson-like events according to sex and age. Based on these reports, the prevalence of the disease notably increases with advancing age. Studies have shown that for individuals aged ≥65 years, the annual incidence rate reaches approximately 0.16%, a tenfold increase, while the prevalence rate is considerably higher, at approximately 0.95%. Conversely, the incidence rate among those under the age of 40 years is exceedingly rare. Furthermore, research into sex disparities suggests that men typically exhibit higher incidence rates than women. However, the prevalence does not consistently show such sex-specific trends, which may be attributed to the generally shorter life expectancy of men [7]. In contrast, some reports have indicated that in Japan, both incidence and prevalence rates are higher among women [8]. These findings are corroborated by the data presented in Table 1. These results not only corroborate the increased incidence of Parkinson’s disease with advancing age but also imply a greater predisposition for Parkinsonian manifestations under the pharmacological influence. Rajput et al. proposed that although intact dopaminergic neurons remain unaffected upon levodopa administration, those compromised may exhibit an elevated risk of toxicity [9]. Furthermore, Brewer et al. demonstrated that neuronal cells from aged rats are more susceptible to lactate, glutamate, and β-amyloid toxicity than their younger counterparts [10], suggesting that senescence confers increased vulnerability to drug-induced neurodegenerative conditions.

### 3.2. Relationship between Parkinson-like Events and Drugs

In our study, we analyzed the relationship between the use of certain medications and the onset of Parkinson-like events and identified 161 drugs that showed statistically significant results. These drugs indicated an increased risk of side effects as denoted by a positive natural logarithm of the odds ratio (ln ROR). The drugs situated in the upper right quadrant of the analysis plot encompass various categories, including 31 treatments for schizophrenia, 15 antidepressants, 14 sleep and sedative agents, and 8 urological drugs. Moreover, we identified 14 drugs classified as high-risk based on the rigorous criteria of an ln ROR > 0 and a −log(*p*-value) > 30, suggesting a significant probability of inducing serious side effects in Parkinson’s disease [4]. This high-risk category includes seven schizophrenia medications, antidepressants, antiepileptic drugs, and sleeping pills, which are referenced in the literature concerning severe adverse effects associated with Parkinson’s disease. These medications are noted for their mechanisms of action, not only affecting dopamine D2 receptors but also targeting GABA and serotonin receptors, highlighting their extensive pharmacological impact. Note that these drugs are not listed in the reports of serious adverse reactions. It is important to highlight that the group includes benzodiazepines, particularly mirtazapine, clonazepam, and flunitrazepam, which are not commonly reported as agents that cause severe side effects. Parkinson’s disease predominantly presents with motor symptoms; however, its impact extends far beyond mere motor function, encompassing various non-motor symptoms. Pathologically, its ramifications reach beyond the dopamine neurons of the nigrostriatal system, affecting other crucial neurotransmitter systems, such as noradrenaline, serotonin, and acetylcholine [11]. Cremer et al. used mouse models lacking the Pitx3 gene, which exhibited a significant and severe loss of dopaminergic neurons in the substantia nigra, and revealed substantial alterations in GABA receptors across various brain regions. These changes coincide with a marked increase in benzodiazepine-binding sites and a simultaneous decrease in striatal nicotinic acetylcholine and serotonergic receptor density [12]. Furthermore, investigations by Huot et al. have underscored the intricate involvement of the serotonergic system in both motor and non-motor symptoms and elucidated complex interactions that contribute to the disease’s multifaceted nature [13]. In alignment with these findings, Rinne et al. documented notable changes in GABA receptor binding within the brains of patients with Parkinson’s disease and observed decreased binding in the substantia nigra [14]. Such insights suggest that Parkinson-like events are not solely attributed to D2 blockade but could also be influenced by medications targeting GABA and serotonin receptors within the substantia nigra.

### 3.3. Hierarchical Clustering Analysis of Side Effects Associated with Parkinsonian-like Events

Hierarchical clustering, a method for grouping similar data points, was used to categorize the side effects associated with Parkinson-like events into three clusters. They were divided into hypertonia, resting tremors, Parkinsonism, Parkinson’s disease, the on–off phenomenon, Parkinsonian gait, muscle rigidity, bradykinesia, and akinesia. Upon visualization using a star dendrogram, it became apparent that muscle hypertonia and resting tremors were notably segregated and positioned at the opposite end of the spectrum from the other side effects. Conversely, muscle rigidity, bradykinesia, and akinesia were clustered together, indicating a shared mechanism underlying bradykinesia. Zaidel et al. noted that the subthalamic nucleus in patients with Parkinson’s disease exhibits oscillatory activity in the beta frequency range (approximately 15 Hz). Interestingly, tremors in Parkinson’s disease did not show a strict correlation with abnormal synchronized oscillations in the basal ganglia. Conversely, akinesia and rigidity demonstrated a stronger correlation with beta oscillations in the basal ganglia [15]. In Parkinson’s disease, tremors are believed to result from increased interactions between the basal ganglia and cerebello-thalamo-cortical circuits, representing a neural mechanism different from that in bradykinesia and muscle rigidity [16,17]. Fearon et al. reported that hypertonia and rigidity originate from different anatomical pathways: rigidity in Parkinson’s disease is primarily caused by dysfunction in the extrapyramidal system [18]. Symptoms, such as muscle rigidity, akinesia, resting tremors, and muscle hypertonia, in Parkinson’s disease, while having some common mechanisms, are not entirely the same because of potentially different brain regions being involved in the onset of the disease.

### 3.4. PCA of Side Effects Related to Parkinson-like Events

PCA was performed to elucidate the characteristics of the side effects. The analysis revealed that the first principal component accounted for 97.4% of the variance, whereas the second principal component contributed 1.35%. A scatter plot was generated using the first and second principal components, with each adverse event depicted as a loading vector. The X-axis represented the first principal component, which was positively correlated with the natural logarithm of the ln ROR for Parkinson-like events. The ln ROR of the side effects associated with Parkinson-like events was designated as variable X, and the value of the first principal component was designated as variable Y. After conducting the bivariate analysis, the R-squared value was calculated to assess the degree of linear association between these variables. Table 3 presents the correlation between the first principal component and ln ROR for Parkinson-like events. The Y-axis represents the first principal component, which was positively correlated with the natural logarithm of the ln ROR for each side effect associated with Parkinson-like events.

In the loading plot, the side effects are notably clustered in the first principal component and closely distributed in a singular direction. This pattern suggests that the mechanisms underlying Parkinson-like events exhibit a high degree of similarity. Further analysis via the score plot reveals that the 14 high-risk drugs, particularly sulpiride and risperidone, are predominantly positioned at the upper end, indicating a frequent reporting of drugs with D2 receptor-blocking effects. This observation aligns with the data presented in Table 3, which confirm that Parkinsonism is frequently reported as a side effect. Despite a potential reporting bias as highlighted by PMDA’s publication of “serious side effect information”, these findings illustrate a consistent pattern among all the side effects. The data suggest that most side effects are likely due to a common mechanism, although the specific sites of receptor inhibition vary across different drugs, leading to variations in the observed side effects. The functions of serotonin 2A receptors and dopamine D2 receptors are intricately associated with both the physiological and pathological aspects of neuropsychiatric disorders [19]. Furthermore, GABA receptor functionality is crucial in the pathology of extrapyramidal disorders and plays a central role in the onset of both drug-induced and disease-related movement disorders [20]. It is likely that the mechanisms underlying the Parkinsonian-like events triggered by these drugs are similar.

### 3.5. Complex Parkinson-like Events Caused by Drugs

The pathogenesis of drug-induced Parkinson-like events is commonly attributed to the blockade of D2 dopamine receptors. However, the intricate underpinnings of each adverse effect remain elusive, with reports scarcely addressing the potential involvement of serotonergic or GABAergic systems. This oversight suggests that the etiology of such events extends beyond mere D2 receptor antagonism, implicating intricate interactions with serotonin and GABA receptors. In this context, Lee et al. confirmed that antagonists targeting serotonin receptors may disrupt the delicate equilibrium between serotonergic and dopaminergic neurotransmissions, with consequential implications for motor function regulation [21]. Moreover, Keller et al. reported that benzodiazepines, including clonazepam and flunitrazepam, when co-administered with antipsychotic drugs, such as haloperidol, chlorpromazine, and clozapine, potentiate the enduring suppression of dopaminergic activity in the midbrain [22]. Therefore, Parkinson-like events are not singularly precipitated by pharmacodynamic actions on dopaminergic pathways but are the result of a multifaceted interplay involving drug molecular architecture, interactive pharmacokinetics, receptor affinity, and the intrinsic neurobiological susceptibilities accentuated by senescence. Consequently, consider that individual neurochemical and physiological variances may exert a significant impact on the manifestation and intensity of these complex pharmacological events. DIP is generally known as a side effect caused by D2 blockers; however, this study suggests that Parkinson-like events are not necessarily triggered by a single mechanism. For future research, identifying drug structures that could induce Parkinson-like events to preemptively prevent such side effects is crucial. Furthermore, extensively investigating the impact of drug interactions on side effects using computational scientific methods is essential. This study has also highlighted specific drug groups and trends in side effects. Particular attention should be paid to drugs such as blonanserin, paroxetine hydrochloride, and benzodiazepines, including mirtazapine, clonazepam, and flunitrazepam, which have been identified as high-risk but not reported for serious side effects, to ensure that the initial symptoms of Parkinson-like events are not overlooked. Moreover, considering the increased susceptibility of elderly patients to such events because of neurological fragility, being particularly vigilant about the drugs most frequently reported from the 161 identified as potentially risky is advisable.

### 3.6. Limitations

This study has several limitations [23]. First, in this study, the database used inherently involves certain issues because of its reliance on self-reported data. In particular, mild symptoms are typically underreported, whereas severe adverse effects are more likely to be reported because of the spontaneous nature of the submission [24]. This setup can lead to reporting bias, a common issue in self-reporting databases. Second, ascertaining the total number of patients who used the drugs included in this study was impossible, which impedes an accurate assessment of adverse events. To enhance the use of our analysis despite these limitations, we implemented filters on the number of reports and avoided the simplistic comparisons of *p*-values and ROR. Third, the JADER database occasionally suffers from missing data, particularly lacking information on sex and age, which can affect the accuracy of our analysis. Consequently, records with such missing data were excluded from our analysis. Moreover, when multiple drugs are administered concurrently, pinpointing the specific cause of adverse events becomes challenging. Although fatal adverse events reported in the JADER database are verified by the PMDA, other adverse events are based on the reporter’s judgment, which may include both actual and questionable adverse events. Despite these issues, the JADER database remains the largest database for voluntary adverse drug reaction reports in Japan and provides valuable insights into drug pharmacology, pharmacokinetics, prescribing patterns, and usage conditions. Furthermore, retrospective analysis of data and signal detection is fundamentally an approach for hypothesis generation, rather than providing prove on side effects, tolerability, or efficacy of an intervention. However, with more and more large datasets becoming readily available, we anticipate that the examination of real-world data, like the one we have provided here, will become even more relevant to drive medical research and progress. Finally, this analysis is confined to a risk assessment of patients who reported adverse reactions in Japan. Consequently, the results may not be generalizable to the broader population. Further detailed studies that account for differences in patient demographics, such as race and medical history, are necessary.

## 4. Materials and Methods

### 4.1. Database (JADER)

The JADER database is an adverse drug reaction database published by PMDA that summarizes suspected adverse drug reactions reported by manufacturers, distributors, or medical institutions. The data used in this study were downloaded from the PMDA website [25], ranging from 1 April 2004, to 31 July 2022. Because this study used anonymized data from an open-access database, the requirements for ethical approval and informed consent were waived by the Ethics Committee of Meiji Pharmaceutical University.

### 4.2. Drugs to Be Analyzed and Adverse Event Terms

The drugs analyzed were those whose side effects are associated with Parkinson-like events. Parkinson-like events were defined by MedDRA/J version 25.0 [3]. Of the 18 narrow-scope terms of standardized MedDRA queries, nine preferred terms with more than 50 reports were analyzed (Table 4).

### 4.3. Creation of a Data Table for Analysis

The analysis was performed using the DRUG, REAC, and DEMO tables of the JADER database. The completed tables were used for analysis. Duplicate data were deleted in each of the three tables and joined by an identification number. Patients taking medications for Parkinson’s disease were excluded from the data tables.

### 4.4. Parkinson-like Events and Patient Characteristics

Epidemiological studies on Parkinson’s disease indicate that the annual incidence rate for individuals aged ≥65 years is approximately 0.16%, whereas it is extremely low for those aged <40 years [6]. Therefore, an analysis was performed in two segments: age 40 years, which is generally considered to have a sharply increased risk of Parkinson’s disease, and age ≥65 years, defined as elderly by the World Health Organization (in the 70s for age-specific reporting in the JADER database). Age was divided into groups according to age. “Adults” was defined as “age under 40 years old”. Elderly individuals in their 70s, 80s, and 90s were defined as “age 70 and older”. Furthermore, individuals in their 40s, 50s, 60s, 70s, 80s,90s, and the elderly were defined as “age 40 and older”. They were further divided into two groups according to the presence or absence of Parkinson-like events, and *p*-values were calculated using a bivariate relationship to examine the presence of a significant difference. The *p*-value was calculated using Fisher’s exact test. The analysis was performed only on data that did not include missing values, unknowns, fetuses, or trimesters.

### 4.5. Relationship between Parkinson-like Events and Drugs

The association between drugs and Parkinson-like events was analyzed. The risk of drug-induced Parkinson-like events was assessed using ROR and Fisher’s exact test. First, a crosstabulation table (Table 5) was created. The cells with zero cannot be calculated. Therefore, 0.5 was added to all the cells as a correction (the Haldane correction) [26]. Drugs with an ROR of ≥1 and a *p*-value of <0.05 in Fisher’s exact test were considered drugs inducing Parkinson-like events [27]. Next, to visualize adverse events, a scatterplot containing ROR and *p*-values was created. This scatterplot used ROR as the natural logarithm (ln ROR) and the *p*-value obtained from Fisher’s exact test as the common logarithm of the reciprocal. Scatterplots correspond to volcano plots and are often used to visualize gene expression trends [28,29].

### 4.6. Hierarchical Clustering

Out of the 18 narrow-scope terms in the standardized MedDRA query, 9 terms that were prioritized based on having 50 or more reports were analyzed (Table 4). We calculated the ROR from the crosstabulation table (Table 5), converted the resulting ROR to the natural logarithm, and performed hierarchical clustering to objectively classify them. The Ward method based on Euclidean distance with loads from the nine preferred terms was used [30]. This method forms clusters incrementally based on data similarity and performs clustering by selecting combinations that minimize the increase in total intra-cluster variance. Therefore, the clusters that are linked in the dendrogram are considered similar.

### 4.7. PCA

The ROR was calculated from the crosstabulation table (Table 5), and it was converted to a natural logarithm. PCA was performed using the correlation matrix and focused on the principal components 1 and 2. PCA is a method that aggregates multivariate data along the direction of maximum variance, using these directions as coordinate axes (principal components). The larger the eigenvalue, which indicates the variance of data for each principal component, the higher the contribution of that principal component to the information. Therefore, it can be said that the characteristics of multivariate data are represented in the two-dimensional space of the X-axis and Y-axis. The closer the distributions are to each other, the more similar the trends that can be observed.

### 4.8. Statistical Analysis

Statistical analyses were performed using the JMP Pro16 software (SAS Institute Inc., Cary, NC, USA). The level of statistical significance was set at 0.05.

## 5. Conclusions

Our research elucidates that the pathogenesis of Parkinson-like events extends beyond the scope of D2 receptor antagonism, indicating that various pharmacological agents can provoke such adverse events through complex, multidimensional mechanisms. The study identifies two primary facets: although the mechanisms of the side effect profile encompassed by Parkinson-like events generally show a pattern of similarity, it is crucial to note the significant variations in the side effects that arise from the differences in receptor binding sites; and the intricate involvement of serotonin and GABA receptors. These findings suggest that the molecular configurations of compounds, drug–drug interactions, receptor affinities, and the neurochemical and physiological diversities that accrue with aging may substantially affect the manifestation of side effects. The inherent complexity in these interactions underscores the potential for delayed recognition or oversight of adverse drug reactions. In summary, our study yielded two main findings. First, Parkinson-like events are induced by a wide range of drugs and are not limited to D2 blockers. Second, clustering methods are useful for classifying the occurrence of Parkinson-like events, and they revealed clear differences in the pathological trends in side effects, suggesting the involvement of different areas of the brain during their onset.

Consequently, our findings underscore the imperative to swiftly identify suspect pharmaceuticals and to refine risk management protocols, thereby enhancing the safety profile of therapeutic interventions.

## Figures and Tables

**Table 1:** Comparison of characteristics between patients with and without Parkinson-like events.

	Patient Background	Parkinson-like Events (1472)	Non-Parkinson-like Events (695,733)	Reporting Odds Ratio	95% Confidence Interval	*p*-Value (Fisher’s Exact Test)
Sex	Female	812/1472	340,077/695,733	1.287	1.161–1.426	<0.0001
	Male	660/1472	355,656/695,733			
Age	≥40 years old	1307/1472	581,156/695,733	1.562	1.328–1.836	<0.0001
	<40 years old	165/1472	114,577/695,733			0.0597
	≥70 years old	761/1472	287,019/695,733	1.524	1.376–1.688	<0.0001
	<70 years old	711/1472	408,714/695,733			0.8218

**Table 2:** Estimated risk of Parkinson-like events with high-risk drugs (odds ratios).

Drugs	Reporting Odds Ratio	95% Confidence Interval	*p*-Value	a + b
SULPIRIDE	20.10	17.87~22.6	<0.0001	10,814
PEROSPIRONE HYDROCHLORIDE	14.48	10.95~19.14	<0.0001	2475
BLONANSERIN	11.13	8.37~14.78	<0.0001	3078
ARIPIPRAZOLE	10.44	8.88~12.28	<0.0001	10,268
HALOPERIDOL	9.37	7.75~11.32	<0.0001	8280
RISPERIDONE	8.50	7.38~9.79	<0.0001	16,740
BIPERIDEN HYDROCHLORIDE	7.16	5.75~8.91	<0.0001	8016
MIRTAZAPINE	6.90	5.39~8.84	<0.0001	6466
QUETIAPINE FUMARATE	6.68	5.63~7.93	<0.0001	14,234
PAROXETINE HYDROCHLORIDE	6.22	5.19~7.47	<0.0001	13,431
OLANZAPINE	5.86	4.81~7.13	<0.0001	12,077
LITHIUM CARBONATE	5.77	4.66~7.13	<0.0001	10,542
CLONAZEPAM	5.56	4.56~6.79	<0.0001	12,455
FLUNITRAZEPAM	5.03	4.28~5.92	<0.0001	20,815

**Table 3:** Correlation between the first principal component and ln ROR in Parkinson-like events.

Preferred Terms	R^2^
PARKINSONISM	0.724
PARKINSON’S DISEASE	0.882
MUSCLE RIGIDITY	0.926
AKINESIA	0.966
BRADYKINESIA	0.958
ON AND OFF PHENOMENON	0.963
PARKINSONIAN GAIT	0.971
HYPERTONIA	0.961
RESTING TREMOR	0.957

**Table 4:** Analysis of nine preferred terms (MedDRA/J version 25.0).

Preferred Terms	Number of Reports
PARKINSONISM	5007
PARKINSON’S DISEASE	1891
MUSCLE RIGIDITY	923
AKINESIA	482
BRADYKINESIA	480
ON AND OFF PHENOMENON	187
PARKINSONIAN GAIT	183
HYPERTONIA	165
RESTING TREMOR	63

**Table 5:** A crosstabulation table and ROR formulas. A crosstabulation table consisting of reports with the suspected drugs, all other reports, reports of Parkinson-like events, and reports of non-Parkinson-like events.

	Parkinson-like Events	Non-Parkinson-like Events
Reports with the suspected drugs	a	b
All other reports	c	d

ROR (Reporting Odds Ratio) = (a/b)/(c/d) = ad/bc.

## Data Availability

Data are contained within the article.

## Supplementary Materials

The following supporting information can be downloaded at: https://www.mdpi.com/article/10.3390/ph17081099/s1, Table S1: Reported odds ratios of drugs for each side effect associated with Parkinson-like events.
